# Over-expression of histone H3K4 demethylase gene *JMJ15* enhances salt tolerance in *Arabidopsis*

**DOI:** 10.3389/fpls.2014.00290

**Published:** 2014-06-24

**Authors:** Yuan Shen, Natalia Conde e Silva, Laure Audonnet, Caroline Servet, Wei Wei, Dao-Xiu Zhou

**Affiliations:** ^1^Saclay Plant Science, Institut de Biologie des Plantes, Université Paris-Sud 11Orsay, France; ^2^UMR 8618, CNRSOrsay, France; ^3^Interdisciplinary Scientific Research Institute, Jianghan UniversityWuhan, China

**Keywords:** histone methylation, jumonji demethylase, JMJ15, abiotic stress tolerance gene, epigenetic regulation, H3K4me3, chromatin modification

## Abstract

Histone H3 lysine 4 trimethylation (H3K4me3) has been shown to be involved in stress-responsive gene expression and gene priming in plants. However, the role of H3K4me3 resetting in the processes is not clear. In this work we studied the expression and function of Arabidopsis H3K4 demethylase gene *JMJ15*. We show that the expression of *JMJ15* was relatively low and was limited to a number of tissues during vegetative growth but was higher in young floral organs. Over-expression of the gene in gain-of-function mutants reduced the plant height with accumulation of lignin in stems, while the loss-of-function mutation did not produce any visible phenotype. The gain-of-function mutants showed enhanced salt tolerance, whereas the loss-of-function mutant was more sensitive to salt compared to the wild type. Transcriptomic analysis revealed that over-expression of *JMJ15* down-regulated many genes which are preferentially marked by H3K4me3 and H3K4me2. Many of the down-regulated genes encode transcription regulators involved in stress responses. The data suggest that increased JMJ15 levels may regulate the gene expression program that enhances stress tolerance.

## Introduction

Histone lysine methylation is an important epigenetic modification for gene expression in eukaryotic cells (Martin and Zhang, 2005; Kouzarides, 2007). Genome-wide analysis has revealed that about two-thirds of Arabidopsis genes are marked by mono-, di- or tri-methylation of histone H3 at residue lysine 4 (H3K4me1/2/3) (Zhang et al., 2009). H3K4me3 is predominantly found at the promoter and 5′ end of genes and is strongly associated with transcriptional activation in plants. H3K4me3 level was found to be increased on responsive genes upon stress treatment, but in some cases the increase was found to be lagged behind gene activation (Kim et al., 2008; Hu et al., 2011), suggesting that H3K4me3 may have a function to mark the active gene state. In addition, H3K4me3 in gene body has been suggested to play a role in transcriptional memory of stress-responsive genes in Arabidopsis (Alvarez-Venegas et al., 2007; Jaskiewicz et al., 2011). Recent results indicated that the H3K4me3 level in gene body was decreased after stress recovery but remained higher than basal state, suggesting that a regulated resetting mechanism is involved in partial removal of H3K4me3 and that remaining H3K4me3 may contribute to the transcriptional memory in *Arabidopsis* (Ding et al., 2012; Kim et al., 2012).

Histone methylation marks are established by evolutionarily conserved SET-domain proteins (named after 3 *Drosophila* genes: *S*u(var)3–9, *E*nhancer of zeste and *T*rithorax). H3K4 methylation is mediated by the Trithorax group proteins (TRX). Arabidopsis Trithorax ATX1 and ATX2 respectively trimethylate and dimethylate H3K4 (Saleh et al., 2008). ATX1 was found to be necessary for stress-induced gene expression (Alvarez-Venegas and Avramova, 2005; Alvarez-Venegas et al., 2007; Ding et al., 2011). Other SET-domain genes (SDG) such as SDG4 and SDG2 are also involved in H3K4 methylation and control of many aspects of plant development (Cartagena et al., 2008; Berr et al., 2010; Guo et al., 2010).

Histone methylation is reversed by histone demethylases. Lysine Specific Demethylase 1 (LSD1) is the first identified histone demethylase to remove mono- and di-methyl groups from H3K4 (Shi et al., 2004). In Arabidopsis there are 4 LSD1-like genes including *FLOWERING LOCUS D* (*FLD*), *LSD1-LIKE 1* (*LDL1*), and *LSD1-LIKE 2* (*LDL2*) that are shown to be involved in flowering time control (Jiang et al., 2007). The second class of histone demethylases that contain the jumonji C (JmjC) domain catalyze histone lysine demethylation through a ferrous ion (Fe(II)) and α-ketoglutaric acid (α-KG)-dependent oxidative reaction (Tsukada et al., 2006). Multiple JmjC domain-containing histone demethylases are identified in animal cells, which are divided into distinct groups including JARID/KDM5, JMJD1/JHDM2/KDM3, JMJD2/KDM4, JMJD3/UTX/KDM6, JHDM1/FBX/KDM2 and the “JmjC domain-only” group. Members of each group target to specific histone lysine residues at different methylation states (Klose et al., 2006). About 20 JmjC domain-containing protein genes are found in Arabidopsis (Lu et al., 2008; Sun and Zhou, 2008; Chen et al., 2011). Most animal and plant JmjC proteins are conserved, while some animal proteins, such as the JMJD3/UTX/KDM6 group that has the H3K27 demethylase activity is not found in plants. Recent data have shown that plant JMJD2/KDM4 homologs can demethylate H3K27 (Lu et al., 2011; Li et al., 2013).

The JARID/KDM5 group catalyzes H3K4me2/3 demethylation in mammalian cells. *Arabidopsis* genome has one JARID/KDM5-like gene (*JMJ17*), whose function is presently unknown. There is a specific group in plants which includes Arabidopsis JMJ14, JMJ15, JMJ16, JMJ18, and JMJ19. The JmjC domains of this group are more closely related to that of the JARID, but structurally similar to that of JMJD2/KDM4 (Chen et al., 2013). JMJ14, JMJ15 and JMJ18 have been reported to have the H3K4me2/3 demethylase activity and to regulate diverse aspects of chromatin function and plant development (Deleris et al., 2010; Lu et al., 2010; Searle et al., 2010; Le Masson et al., 2012; Yang et al., 2012a,b; Cui et al., 2013). However, the function of these H3K4 demethylases in plant stress tolerance has not been evaluated. In this work we provide evidence that increased expression of *JMJ15* preferentially down-regulates H3K4me2/3-marked stress-related genes and enhances salt stress tolerance.

## Materials and methods

### Plant growth

The *Arabidopsis thaliana* ecotype Columbia (Col-0) was used throughout this study. T-DNA mutant lines *jmj15-1* (GABI_257F10), *jmj15-2* (GABI_876B01) and *jmj15-3* (GABI_663C11) were obtained from the Nottingham *Arabidopsis* Stock Center (NASC) and confirmed by PCR. Seeds were surface-sterilized and plants were grown on 0.5 x Murashige and Skoog (MS) medium after stratification at 4°C for 2 days. Plants were analyzed on plates under long-day (LD, 16 h light/8 h dark) or short-day (SD, 8 h light/16 h dark) photoperiods at 20°C. Ten days after germination, plants were transferred to soil and kept in growth rooms under LD conditions.

To test gene expression in response to salt, experiments were carried out with 8 day-old plants, treated with 0.5 x MS supplemented with or without 100 to 150 mM NaCl for 1-5 h. For germination tests, seeds of wild type and *jmj15* mutants were sown on medium containing 130-150 mM NaCl. Images of the Petri dishes were taken 10 days after germination.

### Constructs and transformation

For the histochemical GUS assay, the 2 kb promoter of *JMJ15* was amplified from wild type genomic DNA using the following primers: 5′-*GGATCC*AGAGCTTGGCCATTTCTTGA-3′ (forward) and 5′-*GGTACC*GCACTGAAAGGCTCCATTG-3′ (reverse). *BamHI* and *KpnI* (underlined) were used for digestions. The *JMJ15* promoter fragment was inserted as translational fusion with the *uidA* gene into the pPR97 vector. To generate the *35S-JMJ15-FLAG-HA* construct, the full length cDNA without the stop codon was amplified from total cDNA isolated from Col-0 plants using primers: *TCTAGA*CCTTTGGGTTTTGTGGAGTG (forward) and *TCTAGA*CCAATTCAAATCAACCCCAAA (reverse). Using *XbaI* site, *JMJ15* cDNA was inserted into the binary vector pFA121, which was modified based on pBI121 and contained 2 × FLAG-HA tag. The *pJMJ15-GUS* and *35S-JMJ15-FLAG-HA* constructs were transformed into Agrobacterium tumefaciens strain GV3101 and then transformed the plants using floral dip method.

### Microarray analysis

Total RNA was extracted from 12 day-old seedling using Trizol (Invitrogen) and cleaned using the RNeasy isolation kit (Qiagen). Hybridization with Affymetrix GeneChip *Arabidopsis* ATH1 Genome Array was performed at CapitalBio Corporation. Wild type and both *jmj15* over-expression alleles were performed in two biological repeats. Gene expression changes between the samples were analyzed by the AffylmGUI package from R software. GO annotation was carried out with the GO terms of the TAIR database (http://arabidopsis.org/tools/bulk/go/index.jsp). The percentage of significantly gene enrichment in each TAIR annotated category was calculated as follows: the number of enriched genes divided by N × 100, where N represents the total number of genes annotated in each category. Significantly enriched genes were subsequently analyzed for their H3K4 methylation levels at epigenomics database (http://epigenomics.mcdb.ucla.edu/H3K4m1m2m3/).

### Real-time PCR

For gene expression analysis, two micrograms of total RNA were reverse transcribed into cDNA by ImPromII reverse transcriptase (Promega). Real-time PCR was performed with the LightCycler® 480 SYBR Green I Master (Roche) on a LightCycler 480 (Roche). At least two biological replicates and two technical repeats for every biological replicate were tested. The primers used in this study are listed in Supplementary Table 1.

### Histochemical GUS and lignin staining

GUS staining was performed as previously described (Bertrand et al., 2003). Briefly, plant samples were fixed with 90% acetone on ice for 20 min and were washed with staining buffer (0.2% Triton X-100, 5 mM potassium ferrocyanide, 5 mM potassium ferricyanide, 100 mM NaH_2_PO4 and 100 mM Na_2_HPO4 pH 7.2). Then the samples were immersed in GUS staining solution with 1 mM X-Gluc and placed under vacuum for 20 min. After incubation at 37°C overnight, the staining solution was removed and samples were cleared by sequential changes of 70% (v/v) ethanol and stored at 4°C.

The histological comparative analysis of inflorescence stems between Col-0 and *jmj15* mutants was done at the stage of newly formed green siliques, about 2 weeks after bolting, when the inflorescence stems of wild type reach 20 cm in height. Cross-sections of the inflorescence stems at the basal end were stained for 3 min in phloroglucinol-HCl reagent (Prolabo, VMR International, France) and then observed in ethanol 100%: HCl 37% (9/1, v/v) using a light microscope (Nikon, MULTIZOOM AZ 100).

## Results

### Expression levels of H3K4 demethylase genes

To investigate whether H3K4 demethylase genes are involved in plant stress responses, we analyzed the mRNA levels of *JMJ14* (At4g20400), *JMJ15* (At2g34880), *JMJ16* (At1g08620)*, JMJ17* (At1g63490)*, JMJ18* (At3g30810), and *JMJ19* (At2g38950) genes in 8 day-old seedlings grown in ½MS media under continuous light, then transferred to 100 mM NaCl or to ½MS solution for 5 hours. In untreated (½MS) seedlings, the expression levels of the 6 genes varied considerably. The relative expression levels of *JMJ17, JMJ18*, and *JMJ19* were much higher (>10^2^) than that of *JMJ14, JMJ15*, and *JMJ16* (Supplementary Figure 1). NaCl treatment did not dramatically affect the expression of these genes, although some decrease of *JMJ14* and *JMJ18* and some increase of *JMJ15* transcript levels were detected.

### JMJ15 displayed a highly tissue-specific expression pattern

**Figure 1:**
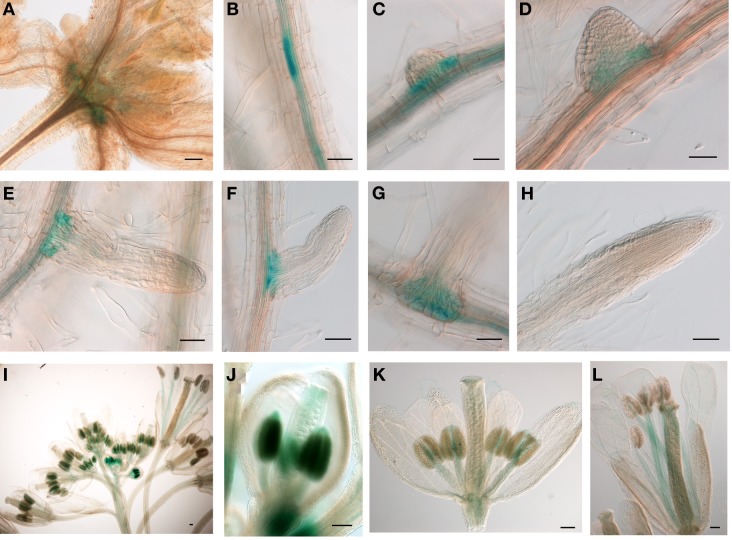
**The 2.0 kb promoter activity of *JMJ15* in transgenic plants**. In seedlings, the GUS activity was detected in the base of rosette leaves **(A)**, root vascular tissues **(B)**, and bases of growing lateral roots **(C–G)**, but not in root tip **(H)**. In flower buds, the GUS activity was detected in anthers and in carpels **(I,J)**, which become weak in opened flowers, but remained in stamen filaments **(K,L)**. Bar = 0.1 mm.

The relatively low expression level of *JMJ15* was in agreement with previous data showing that the 1.5 kb promoter region of *JMJ15* is weak in vegetative tissues (Hong et al., 2009). To study the temporal and spatial expression pattern of *JMJ15*, we used a larger promoter region of *JMJ15* (−2051 to +14 bp relative to ATG) to make a *GUS* reporter translational fusion construct and transformed Arabidopsis Col-0 plants. Three independent GUS reporter lines were characterized. All showed a similar pattern of GUS expression. In seedlings, GUS activity was detected only at the base of rosette leaves and root vascular tissues (Figures 1A–C). Interestingly, a higher accumulation of GUS activity was detected in pericycle cells that initiated to lateral root meristem (Figures 1B–D). The GUS activity remained to be detected at the base of the growing lateral roots (Figures 1E–G), but not in the root tip (Figure 1H). In the inflorescence, GUS activity was strongly detected in young anthers and was detectable in carpels, but the activities became weaker in the mature flower (Figures 1I–L). This temporal and tissue-specific expression pattern suggested that *JMJ15* may have a function in plant development.

### JMJ15 gain-of-function mutations showed a reduced plant height phenotype

**Figure 2:**
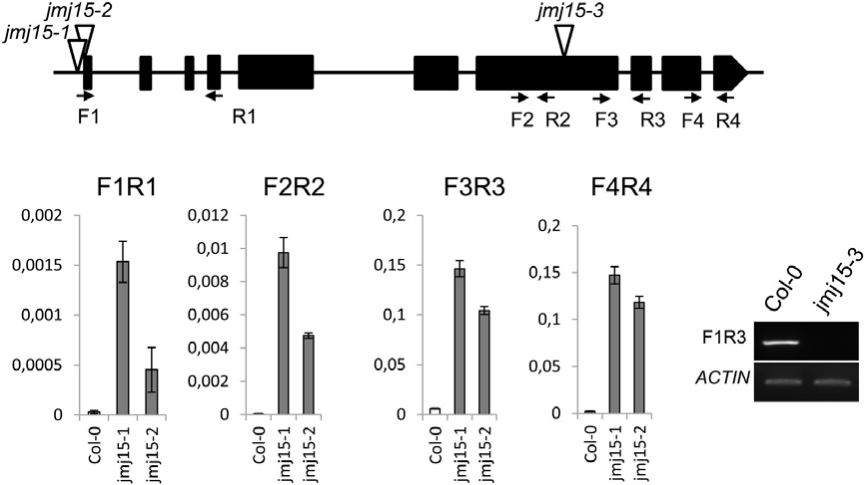
**Characterization of *JMJ15* T-DNA insertion mutants**. The insertion positions of the 3 alleles are indicated by open arrows. The exons are represented by black boxes. The positions of forward (F) and reverse (R) primers are indicated by arrows. The expression levels (relative to *ACTIN2* mRNA) in *jmj15-1* and *jmj15-2* compared to wild type were tested by qRT-PCR using the 4 indicated primer sets. The transcript in *jmj15-3* compared to wild type was analyzed by RT-PCR using the indicated primers.

The *JMJ15* gene contains 10 exons and encodes a polypeptide of 806 amino acids with distinct domains, including the JmjN domain, JmjC domain, a C5HC2 zinc finger, and the FY-rich N-terminus (FYRN)/FY-rich C-terminus (FYRC) domains, which are conserved in JMJ14, JMJ16, and JMJ18 (Supplementary Figure 2) (Lu et al., 2008). To study the function of JMJ15 in gene expression and plant development, we characterized 3 T-DNA insertion mutants: *jmj15-1*(GK-257F10)*, jmj15-2* (GK-876B01) and *jmj15-3* (GK-663C11). In *jmj15-1* and *jmj15-2*, the T-DNA was inserted in the 5′ end, and in *jmj15-3* the T-DNA was inserted in the seventh exon of the gene (Figure 2). RT-PCR analysis with 4 pairs of primers that covered the whole coding region of the gene, revealed that the transcript level of *JMJ15* was dramatically increased in *jmj15-1* and *jmj15-2*, but the transcript was interrupted in *jmj15-3* compared to wild-type (Figure 2). The insertion in *jmj15-1* and *jmj15-2* did not alter the 5′ end of the coding region, as the primer set F1 (that cover the 5′end of the coding region) and R1 successfully amplify the transcripts from the mutants. The data suggested that *jmj15-1* and *jmj15-2* were gain-of-function mutants that overexpressed the gene and that *jmj15-3* was a loss-of-function mutant.

**Figure 3:**
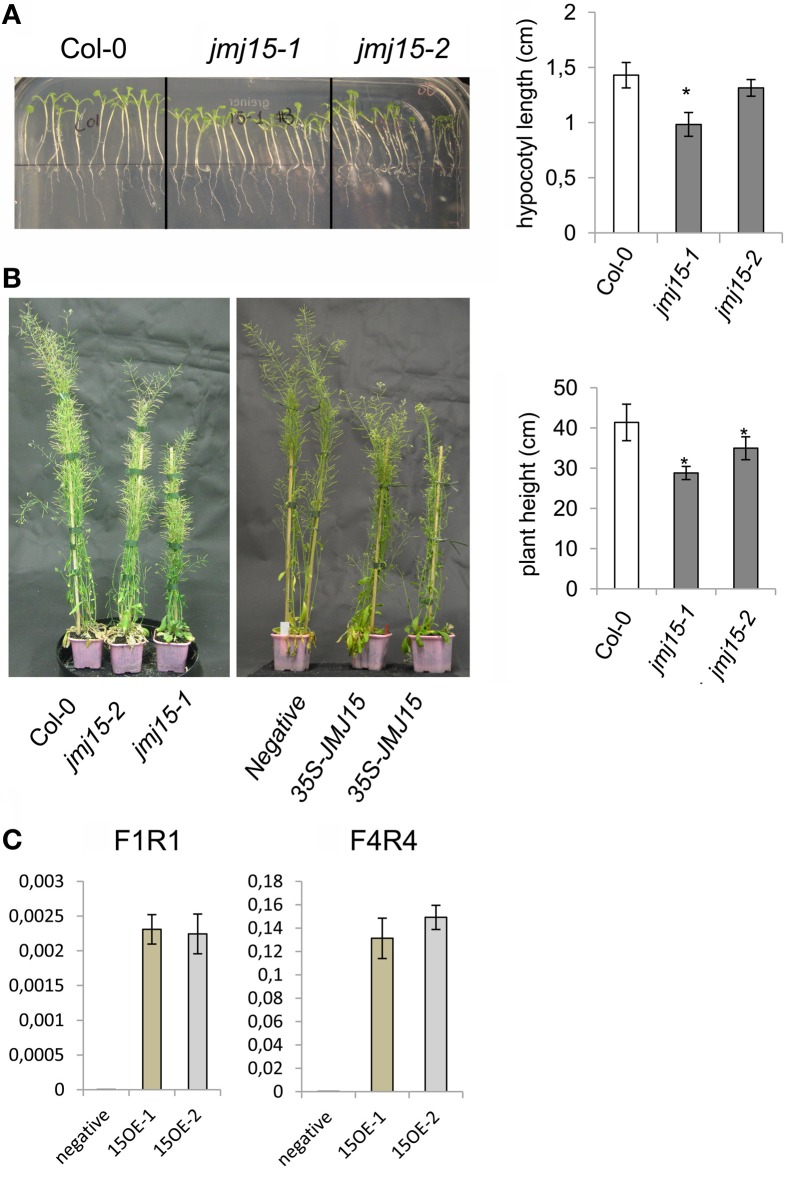
***Jmj15* gain-of-function mutations reduced plant height**. **(A)** Phenotype of hypocotyls of short day-grown (in ½MS medium) seedlings of *jmj15-1* and *jmj15-2* compared to wild type col-0 (left). Lengths of hypocotyls were measured from 30 plants per genotype (right). **(B)** Plant height at mature stage of *jmj15-1* and *jmj15-2* compared to wild type (left) and of 35S-JMJ15-HA over-expression plants compared to negative transgenic plants (middle) grown in soil in greenhouse under long day conditions. Plant heights were measured from 10 plants per genotype (right). Bar = means ± SD. Asterisks indicate the significance of difference from wild type by student *t*-tests (*P* < 0.01). **(C)** *JMJ15* transcript levels (relative to *ACTIN2* transcripts) in over-expression transgenic positive (15OE-1 and 15OE-2) and negative plants.

The *jmj15-3* loss-of-function mutation did not display any visible phenotype in normal growth conditions, confirming previous observations (Yang et al., 2012a). However, in short day (8 h light/16 h dark)-grown seedlings, *jmj15-1* and *jmj15-2* mutants produced slightly shorter hypocotyls compared to wild type (Figure 3A). At the mature stage, the plant height of *jmj15-1* and *jmj15*-2 were clearly reduced compared to wild type (Figure 3B). To study whether the plant height phenotype of *jmj15-1* and *jmj15-2* was due to increased expression of the gene, we made *35S-JMJ15-FLAG-HA* construct and obtained *JMJ15* over-expression transgenic plants. The transgenic plants also displayed the reduced plant height phenotype at mature stage (Figures 3B,C).

**Figure 4:**
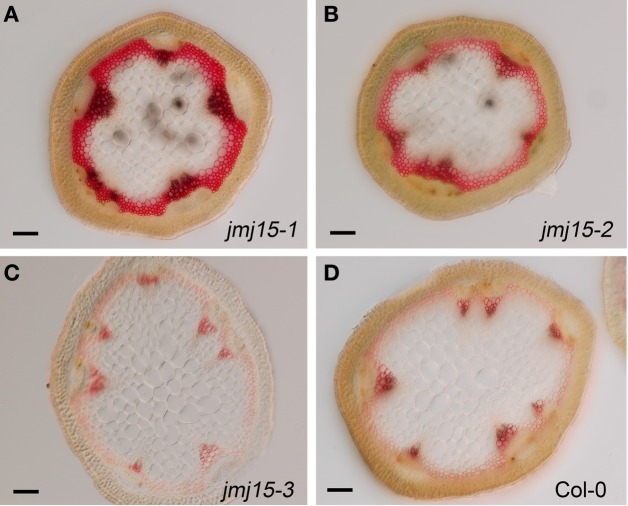
**Lignin accumulation in *jmj15-1, jmj15-2*, and *jmj15-3* mutants compared to wild type**. Sections of the basal part of the inflorescence stem of 5 week-old plants (grown in long day in greenhouse) were stained with phloroglucinol and examined by light microscopy. Phloroglucinol reacts with coniferaldehyde groups in lignin, and the color intensity reflects the total lignin content. Bar = 0.1 mm.

The plant height phenotype of the *jmj15* gain-of-function mutants prompted us to further investigate the stem structure by using histochemical method. Sections of the basal part of the inflorescence stem of 5 week-old plants (grown in long day in greenhouse) were stained with phloroglucinol and examined by light microscopy. Phloroglucinol reacts with coniferaldehyde groups in lignin, and the color intensity reflects the total lignin content. The analysis revealed that *jmj15-1* and *jmj15-2* exhibited a significantly deeper red staining in the stem vascular system and interfascicular fibers compared to that in wild type and *jmj15-3* (Figure 4). This observation suggested that over-expression of *JMJ15* resulted in an increase of the total lignin content in the stems concurrently with stem growth reduction.

### Over-expression of JMJ15 preferentially repressed genes marked by H3K4 methylation

**Figure 5:**
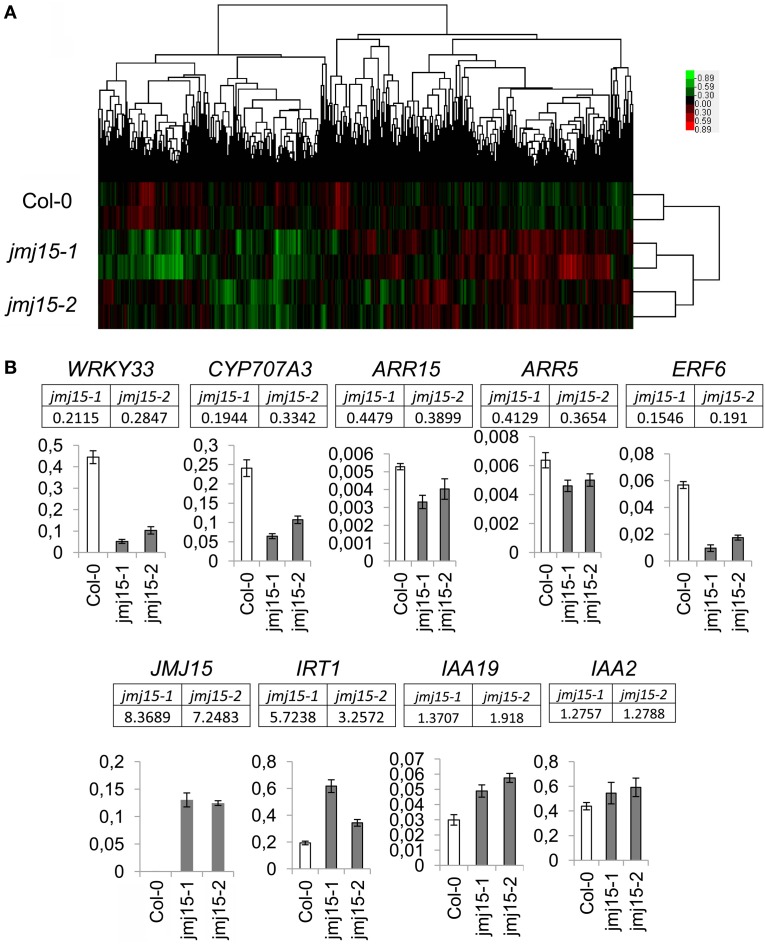
**Transcriptomic analysis of *jmj15-1* and *jmj15-2* (12 day-old) seedlings compared to wild type grown in ½MS medium**. **(A)** Pair-wise plots of the microarray hybridization signals between the biological replicates of each sample and the between wild type and two 2 mutant alleles. Green: down-regulated genes; red: up-regulated genes. **(B)** Five down-regulated and 4 up-regulated genes in the two mutants (microarray signals relative to wild type are indicated below the respective genes) were validated by qRT-PCR. Bar = means ± SD from 3 replicates.

To determine the effect of *JMJ15* over-expression on gene expression, the transcriptomes of *jmj15-1, jmj15-2* and wild type seedlings (12 day-old, grown in ½MS media) were analyzed by using the Affymetrix Arabidopsis ATH1 Genome Array. Two biological replicates for each sample were analyzed. Pair-wise plots of the microarray data revealed a good correlation of the hybridization signals between the biological replicates of each sample and between the 2 mutant alleles (Figure 5A). The average hybridization signals of the replicates of both mutants were normalized and compared with the wild type signals. Up- and down-regulated genes in both *jmj15-1* and *jmj15-2* were filtrated with the threshold >2 fold changes (*p*-value < 0.01) compared to wild type. The analysis revealed 23 up-regulated and 164 down-regulated genes in the mutant lines (Supplementary Dataset 1). In addition, the analysis revealed a much high expression level of *JMJ15* itself (>7-8 folds) in the mutants compared to wild type (Supplementary Dataset 1), confirming the over-expression of the gene in the mutants. The higher number of down-regulated genes compared to up-regulated ones suggested that elevated *JMJ15* expression mainly repressed genes and that JMJ15 acted as a transcriptional repressor, consistent with its H3K4 demethylase activity (Liu et al., 2010; Yang et al., 2012a). To validate the microarray data, we checked 5 down-regulated and 4 up-regulated (including *JMJ15*) genes by RT-qPCR. The relative transcript level changes in the mutants compared to wild type detected by RT-qPCR were in agreement with that from the microarray analysis (Figure 5B).

**Figure 6:**
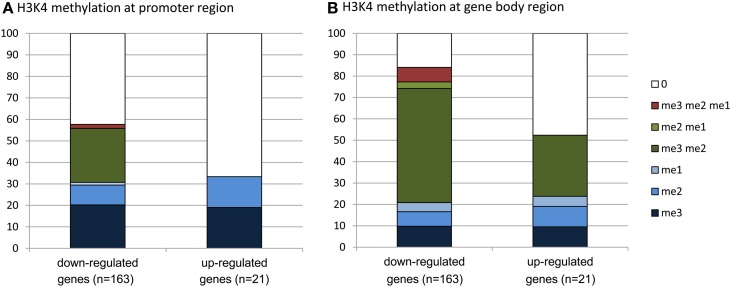
**Down-regulated genes are highly enriched for H3K4me2/3**. Up-regulated (21) and down-regulated (163) genes in *jmj15-1* or *jmj15-2* mutants (changes >2 folds) were compared with genome-wide H3K4 methylation data. Percentages of H3K4me3, H3K4me2, H3K4me1 and their combinations on the promoter **(A)** and the gene body **(B)** regions are presented.

To evaluate whether there was any enrichment of the deregulated genes for H3K4me2/3, we compared the deregulated genes with the genome-wide H3K4me2/3/1 data obtained from wild type seedlings (Zhang et al., 2009). The analysis revealed that about 83% of the down-regulated genes were marked by the H3K4 methylation (mostly by H3K4me2, H3K4me3, or both) in the gene bodies, compared to about 52% of up-regulated genes (Figure 6, Supplementary Dataset 1). About 58% of the down-regulated genes were marked by H3K4me3, H3K4me2, or H3K4me2/3 in the promoter region (in the −500 bp region relative to TSS), compared to about 32% of up-regulated genes. Compared to up-regulated genes, the down-regulated ones were clearly enriched for the H3K4me2/3 double methylation marks. This analysis suggested that JMJ15-mediated gene repression might be achieved by demethylating H3K4 and indicated that JMJ15 preferentially repressed genes that have the H3K4me2/3 double methylation marks.

### Over-expression of JMJ15 preferentially repressed stress regulatory genes

Gene ontology (GO) analysis of the deregulated genes using the GO Slim Classification (http://www.arabidopsis.org/help/helppages/go_slim_help.jsp) revealed that a large proportion of the deregulated genes (23.73%) in the *jmj15* mutants had a function in the nucleus (Supplementary Figure 3A). Interestingly, 36 out of the 164 down-regulated genes were transcriptional regulators (Supplementary Dataset 1). Remarkably, about 50% (82/164) of the down-regulated genes belonged to the stress-responsive categories (Supplementary Figure 3B, Supplementary Dataset 1), suggesting that JMJ15 may have a function in stress-responsive gene expression. We noticed that among the greater than 5 fold down-regulated genes (31), about 50% (15) encode transcription factors (Supplementary Dataset 1). These included the stress-responsive zinc finger protein STZ/Zat10 (At1g27730) (Sakamoto et al., 2000), the stress-responsive WRKY proteins WRKY40 (At1g80840) and WRKY33 (At2g38470) (Jiang and Deyholos, 2009), the cold-responsive factor CBF2 (At4g25470) (Vogel et al., 2005), the ethylene-responsive-element binding proteins ATERF6 (At4g17490) and ATERF11 (At1g28370) (Li et al., 2011; Dubois et al., 2013) (Supplementary Dataset 1). Among them, the decreased expression of *WRKY33* and *ERF6* was validated by RT-qPCR (Figure 5B).

### JMJ15 gain-of-function mutations enhanced salt stress tolerance

**Figure 7:**
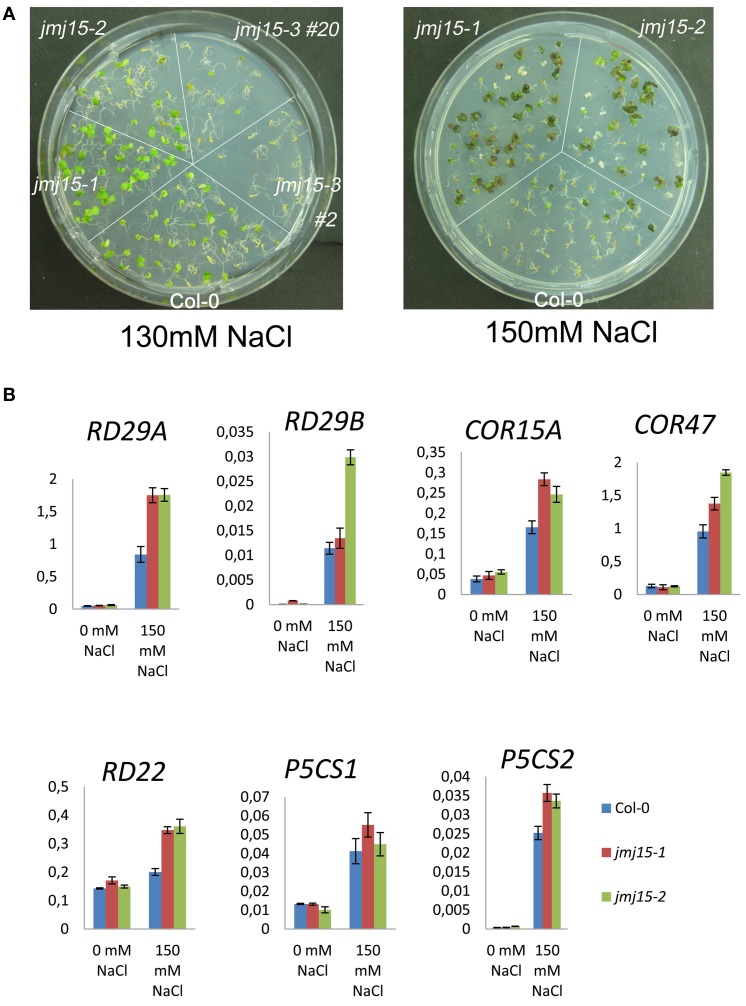
**Comparison of sensitivity to NaCl and stress-responsive gene expression between *jmj15* mutants with wild type**. **(A)** Wild type, *jmj15-1, jmj15-2*, and *jmj15-3* seeds were germinated for 12 days on ½MS supplemented with indicated concentrations of NaCl. Images of the Petri dishes were taken 10 days after germination. **(B)** Transcript levels (relative to *ACTIN2*) of indicated stress-responsive marker genes in wild type and *jmj15-1* and *jmj15-2* mutant seedlings treated with or without 150 mM NaCl for 1 h.

To study whether *jmj15* mutations affected plant tolerance to stress, we germinated seeds of wild type, *jmj15-1, jmj15-2* and *jmj15-3* mutants on ½MS media containing 130 mM or 150 mM NaCl. The seedling growth phenotype shown in Figure 7A indicated that the gain-of-function mutations (*jmj15-1* and *jmj15-2*) enhanced plant tolerance to salt stress, whereas the loss-of-function mutation (*jmj15-3*) reduced the stress resistance. To study whether *JMJ15* over-expression affected stress-responsive gene expression, we analyzed the transcript levels of several stress-responsive marker genes (i.e., *RD29A, RD29B, RD22, COR15A, COR47, P5CS1*, and *P5CS2*) in the gain-of-function mutants grown in normal conditions then treated with or without 150 mM NaCl for 1 h. Without treatment, the expression of these genes was not clearly affected by *JMJ15* over-expression. After 1h treatment with 150 mM NaCl, the expression of the marker genes was induced in both wild type and the gain-of-function mutants, but the induction of *RD29A, RD22*, and *COR15* was clearly higher in the mutants (Figure 7B). The higher induction of the stress-responsive genes might be associated with the enhanced salt tolerance phenotype of the mutants.

## Discussion

### Function of JMJ15 in stress tolerance

In this work we have shown that *JMJ15* displayed a temporal and tissue-specific expression pattern. Constitutive or over-expression of the gene, as a result of T-DNA insertions in the 5′ region, preferentially repressed genes marked by H3K4me2/3, suggesting that JMJ15-mediated gene repression may be achieved mainly through demethylation of H3K4me2/3. The observation that about a third of the down-regulated genes are related to stress implies that JMJ15-dependent H3K4me2/3 levels are important for the expression of this category of genes. This is consistent with numerous observations that H3K4me3 is associated with the induction of biotic and abiotic stress-responsive genes (van Dijk et al., 2010; Hu et al., 2011; Jaskiewicz et al., 2011; Zong et al., 2013; To and Kim, 2014), and that ATX1 that trimethylates H3K4 in the genic region is required for stress-responsive gene expression (Ding et al., 2009, 2011). Microarray analysis of *atx1* mutant seedlings revealed that 424 genes were up-regulated and 328 genes were down-regulated more than 2 folds compared to wild type (Alvarez-Venegas et al., 2006). Similarly, 271 genes were found to be up-regulated and 321 genes down-regulated in mutant seedlings of another H3K4 methyltransferase gene *SDG2* (Guo et al., 2010). However there was no clear correlation between transcription changes in *sdg2* and *atx1* (Guo et al., 2010), suggesting that the two enzymes may regulate different targets. Comparison of the deregulated genes did not reveal a clear overlap, although there was a relatively higher number of overlapped genes between *jmj15* and *atx1* than between *jmj15* and *sdg2* (Supplementary Figure 4). These proteins may targets to different loci. In addition, the present data showing that the mostly down-regulated genes encode transcription factors involved in stress responses raise the hypothesis that *JMJ15* might be a higher hierarchical regulator primarily to regulate stress-responsive gene transcription programs in Arabidopsis. Since *JMJ15* is closely related to *JMJ14, JMJ16*, and *JMJ18* (Lu et al., 2008; Sun and Zhou, 2008), the ectopically expressed *JMJ15* may also regulate the targets of the other related demethylases.

The observations that the *jmj15* gain-of-function mutants showed enhanced salt tolerance at seedling stage and that the loss-of-function mutant was more sensitive to salt stress than wild type, suggest that JMJ15 is required for salt tolerance. The data showing that the gain-of-function mutants displayed reduced growth and increased stem lignification, which are suggested to be associated with stress responses (Moura et al., 2010; Golldack et al., 2013), support the hypothesis that increased JMJ15 levels may regulate the gene expression program that integrates plant growth to stress tolerance. Among the mostly repressed genes in *jmj15* gain-of-function mutants was *STZ/Zat10* that encodes a C2H2-zinc finger protein associated with the ERF amphiphilic repression (EAR) domain (Supplementary Dataset 1). STZ/Zat10 has been shown to be a transcriptional suppressor of stress-responsive genes (Sakamoto et al., 2004). Knockout and RNAi of the gene could enhance plant tolerance to abiotic stress (Mittler et al., 2006). As the STZ locus displays a high level of H3K4me3 (Supplementary Figure 5), it is possible that the repression of *STZ/Zat10* by JMJ15 through H3K4me2/3 demethylation is associated with the enhanced salt tolerance phenotype of the mutants. STZ/Zat10 may be a major player in JMJ15-mediated regulatory network of stress tolerance. However, the observations that several tested stress-responsive marker genes were not changed in the over-expression plants under normal conditions but showed a greater induction during salt stress (Figure 7B), suggest that they might be among the target genes of the transcription repressors ST/Zat10 and AtERF11 under salt stress. However, among the repressed transcription factor genes, some are likely activators (WRKY33). The mechanism of JMJ15-mediated salt tolerance is complex, which may be resulted from a combination of different functions of JMJ15 in gene regulation.

### Developmental function of JMJ15

Consistent with previous results (Yang et al., 2012a), the loss-of-function mutation identified in this study (*jmj15-3*) did not produce any visible phenotype. *JMJ15* was first identified as *Maternal Effect Embryo Arrest 27* (*MEE27*) in a genetic screen for mutants defective in female gametophyte development (Pagnussat et al., 2005). However, no embryonic defect was observed in *jmj15* loss-of-function mutants (Yang et al., 2012a). Either the mutation was compensated by highly expressed homologs (e.g., J*MJ18*, Hong et al., 2009) or JMJ15-dependent H3K4 demethylation is not sufficient to lead to any morphological change. In addition, another study has identified *JMJ15* as a maternally imprinted gene (Hsieh et al., 2011), however, our data showing the high promoter activity of *JMJ15* in anthers do not support that observation.

It is reported that JMJ14 demethylates H3K4me2/3 at the *Flowering Locus T* (*FT*) locus and represses expression of the gene and that *jmj14* loss-of-function mutants display an early flowering phenotype (Jeong et al., 2009; Lu et al., 2010). Conversely, JMJ18 directly binds to and represses the flowering repressor gene, *Flowering Locus C* (*FLC*), through H3K4me2/3 demethylation. Consequently, loss-of-function mutations of *JMJ18* result in a weak late-flowering phenotype, while *JMJ18* overexpressors exhibit an early flowering phenotype (Yang et al., 2012b). These observations support the notion that members of this H3K4 demethylase group target to different loci and have distinct functions in plant development. However, Yang et al have shown that, like *JMJ18, JMJ15* over-expression plants showed repressed *FLC* expression and produced an early flower phenotype (Yang et al., 2012a). But unlike *jmj18* mutants (Yang et al., 2012a), the *jmj15-3* loss-of-function mutation did not alter the flowering phenotype. Possibly, JMJ15 at elevated levels may demethylate and repress genes that normally targeted by JMJ18 in wild type plants. However, the *jmj15-1* and *jmj15-2* gain-of-mutation mutants did not show any clear flowering phenotype. This discrepancy may be due to difference in expression levels or tissue-specificity of JMJ15 in the over-expression plants and the mutant alleles.

### Conflict of interest statement

The authors declare that the research was conducted in the absence of any commercial or financial relationships that could be construed as a potential conflict of interest.
