# Supplementary material for: Over-expression of histone H3K4 demethylase gene JMJ15 enhances salt tolerance in Arabidopsis
Source: Front Plant Sci. 2014 Jun 24;5:290. doi: 10.3389/fpls.2014.00290 (PMC4068201; doi:10.3389/fpls.2014.00290)
Supplement: Supplementary file 1 [file Presentation1.ZIP › Supp Fig 1-5.PDF]

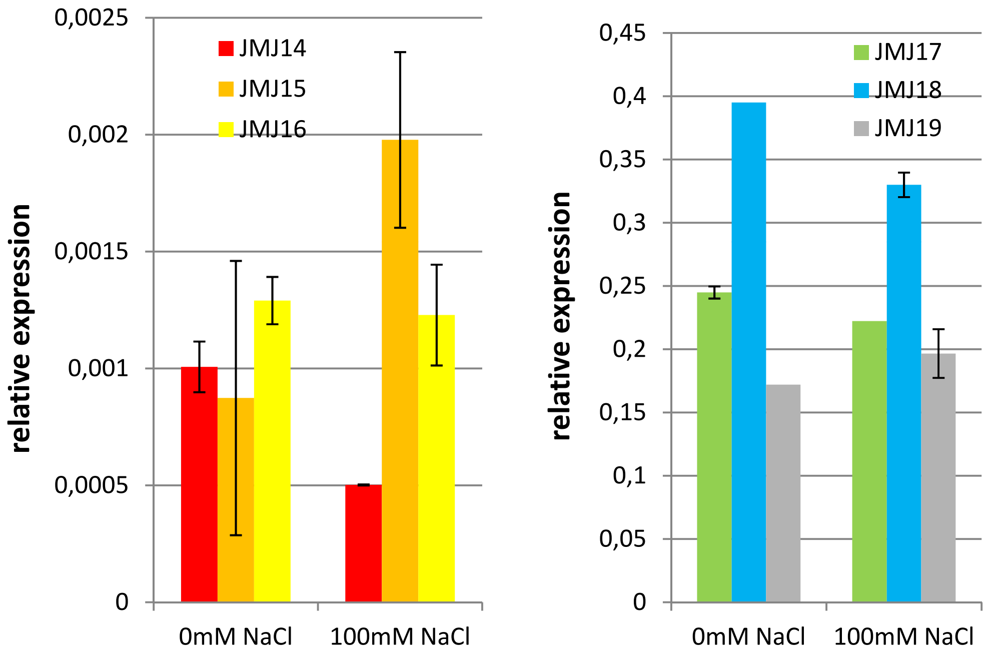

Figure S1. Expression levels of 6 potential H3K4 demethylase genes in Arabidopsis seedlings treated with or without 100 mM NaCl for 5 hours. The transcript levels detected by RT-qPCR are presented as relative to that of the reference gene At4g34270.

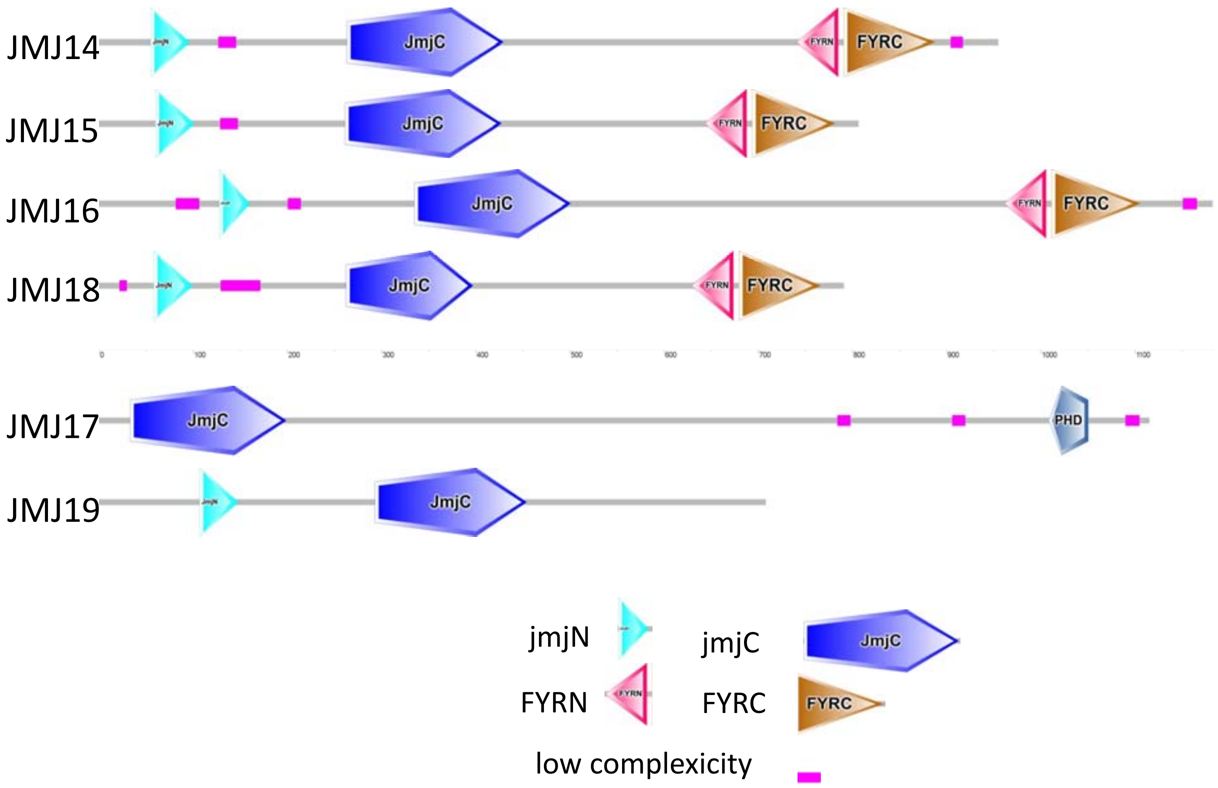

Figure S2. Comparison of protein structures of the 6 JmjC genes specific to plants.

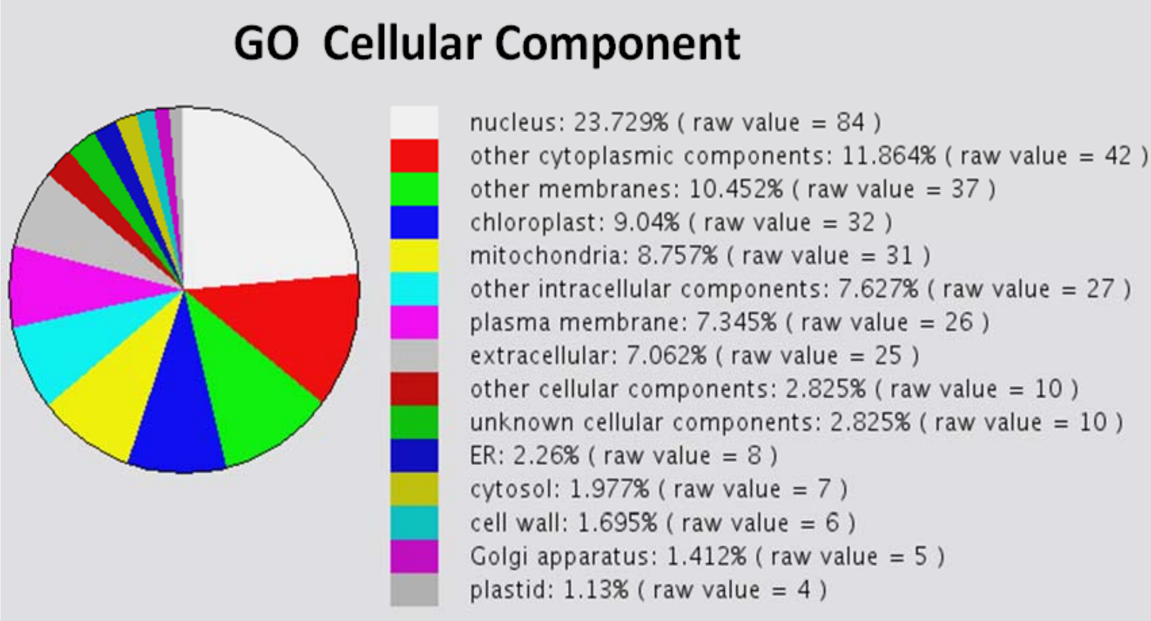

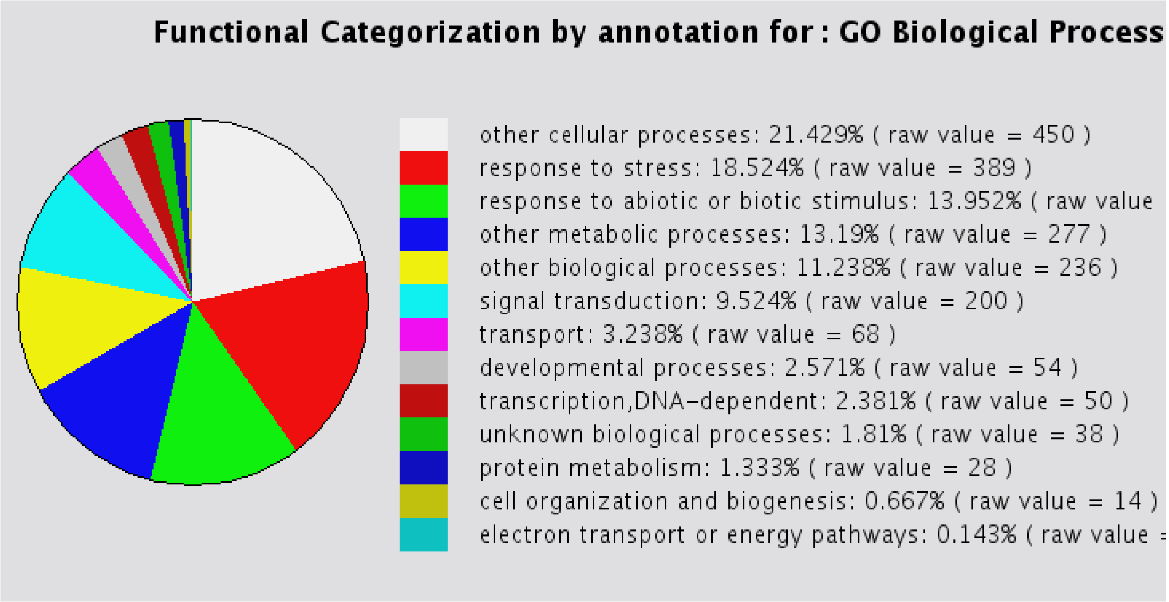

Figure S3. Gene ontology analysis of down-regulated genes in *jmj15* gain-of-function mutants.

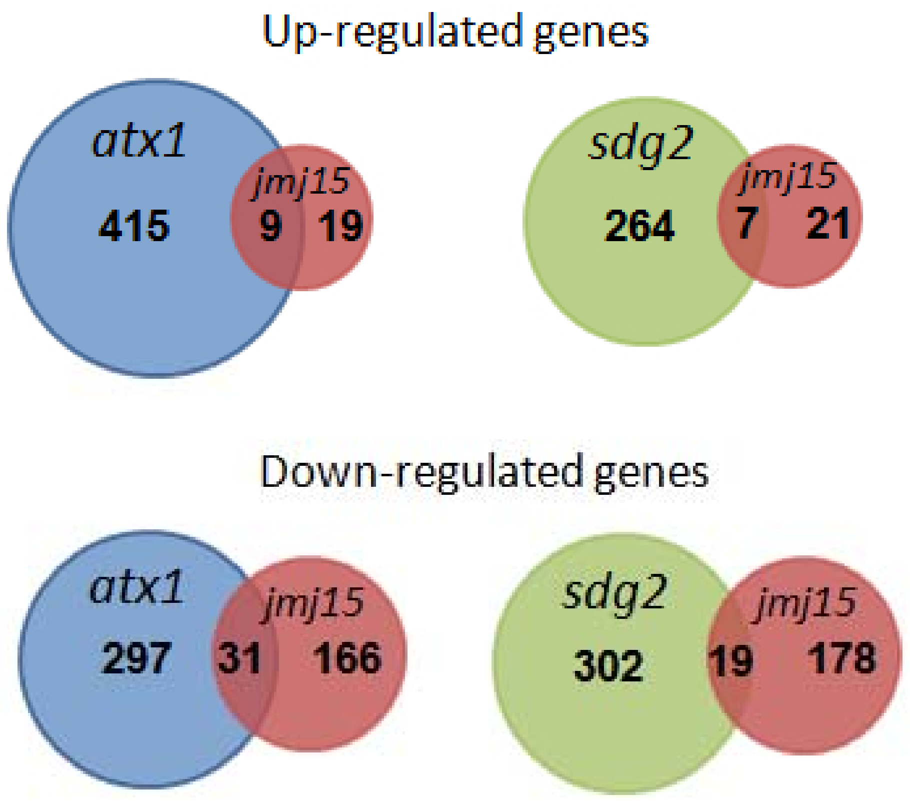

Figure S4. Overlaps of de-regulated genes between *jmj15* gain-of-function mutants and *atx1* or *sdg2* mutants.

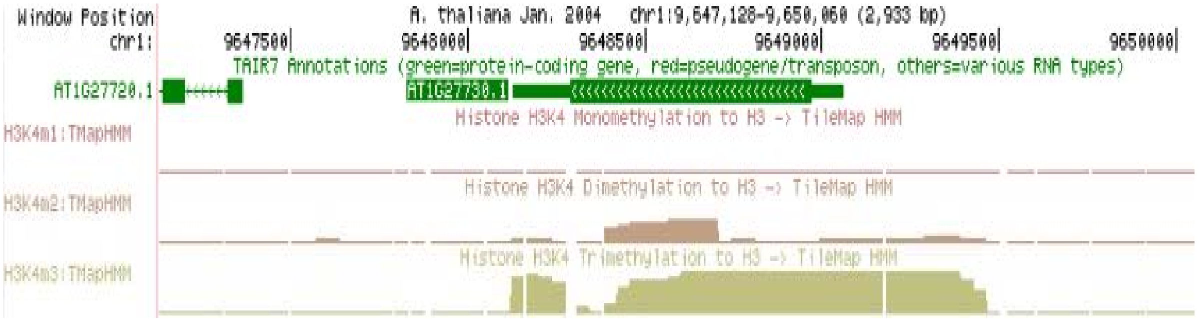

Figure S5. H3K4 methylation on the STZ/Zat10 locus.
