# Supplementary material for: Over-expression of histone H3K4 demethylase gene JMJ15 enhances salt tolerance in Arabidopsis
Source: Front Plant Sci. 2014 Jun 24;5:290. doi: 10.3389/fpls.2014.00290 (PMC4068201; doi:10.3389/fpls.2014.00290)
Supplement: Supplementary file 1 [file Presentation1.ZIP › Supp Table 1.pdf]

Supplementary Table 1. Sequences of the primers used in this study

Gene	Forward (5’→3’)	Reverse (5’→3’)
JMJ15-F1, R1	CCTTTGGGTTTTGTGGAGTG	CACCAATGTCTGGCCTCTTT
JMJ15-F2, R2	CCTTGGAGCAGCTTTTGAAG	AACCATTTCCAAGAGCCTCA
JMJ15-F3, R3	ATCTTGGGTTCTTGGTCGTG	GAAGAGTGGACCCAGAAGTCC
JMJ15-F4, R4	GAGATCATGAGACGCAGCAA	TCATTTCCAGCGAATCCTTC
JMJ14	TGGTTGTTTAGGAGGGAAGG	TGGTTAGGTTTGTTGGGTCA
JMJ16	GGAGATTCGCTGCGTGATAC	CGTGGCTGTTAGTTGCCATT
JMJ17	CATGCAGAGCAGGAACTTCA	GTGGAGTGGTTTCTGCCAGT
JMJ18	GAAATGGTGATGCGGAGAGT	AGTCGATGGTTTGGGTCAAG
JMJ19	AATCGCAAAGGATGGACAAG	CCTCATGTGGTTCAGACACG
WRKY33	AAGGGAAACCCAAATCCAAG	AAGACGAATCCTGTGGTGCT
CYP707A3	AAAGCAGGATTAACCGACGA	TTGCCATTTGCTCTTCAGTG
ARR15	GAAGGAGCAGAGGAGTTTTTG	GTCGTCATCAAGGGAGGAA
ARR5	TGTCCTGATTCTTTCGGCTTACA	AAAAACCCATCTTTGTCACTCTTGA
ERF6	GAATCCTCCTCGCGTTACTG	GCCTCATCCTCACTCCTCTG
IRT1	CTCCAACCAGACGGAAACAT	TAGCGAGAAATCCGGAGAAA
IAA2	CGACGCTCCTGCTCTAGACT	CCCGAAGTTTCGTCTTTCAC
IAA19	GTGATGTACCTTGGGGGATG	TGAACCAGCTCCTTGCTTCT
RD29A	CGGTGGAAGAGGAAGTGAAA	AACCAGCCAGATGATTTTGG
RD29B	TGGTGGGGAAAGTTAAAGGAG	TTCCCAGAATCTTGAACTCCC
RD22	GGATGCGAGCTAAAGCAGTT	ATGAGTCTCCGGGAGGAAGT
COR15A	CAGAAAACTCAGTTCGTCGT	CCGCAGCTTTCTCAGCTTCT
COR47	ACAGAGGTTACGGATCGTGGA	ACTCGAGCGTCGTTGTCTCTT
P5CS1	CGACGGAGACAATGGTGGAATTGT	GCTTGGATGGGAATGTCCTG
P5CS2	CACCCATAAGGATCTTCCTGTCTT	CAATTCTCAACAGCCTCTGTCC
ACTIN2	CGCTGACCGTATGAGCAAAGA	GCAAGAATGGAACCACCGATC
At4g34270	GCACCAACTGTTCTTCGTGA	GGTCGCTCCAGACTGCTAAG
